# Extracellular Vesicles Released by *Leishmania (Leishmania) amazonensis* Promastigotes with Distinct Virulence Profile Differently Modulate the Macrophage Functions

**DOI:** 10.3390/microorganisms11122973

**Published:** 2023-12-13

**Authors:** Rogéria Cristina Zauli, Isabelle Carlos de Souza Perez, Aline Correia Costa de Morais, Ana Carolina Ciaccio, Andrey Sladkevicius Vidal, Rodrigo Pedro Soares, Ana Claudia Torrecilhas, Wagner Luiz Batista, Patricia Xander

**Affiliations:** 1 Programa de Pós-Graduação Biologia-Química, Instituto de Ciências Ambientais Químicas e Farmacêuticas, Universidade Federal de São Paulo, Diadema 04021-001, SP, Brazil; rczauli@unifesp.br (R.C.Z.);; 2 Curso de Ciências Biológicas, Instituto de Ciências Ambientais Químicas e Farmacêuticas, Universidade Federal de São Paulo, Diadema 04021-001, SP, Brazil; 3 Biotecnologia Aplicada a Patógenos (BAP), Instituto René Rachou, Fundação Oswaldo Cruz (FIOCRUZ), Belo Horizonte 30190-002, MG, Brazil; 4 Departamento de Ciências Farmacêuticas, Instituto de Ciências Ambientais Químicas e Farmacêuticas, Universidade Federal de São Paulo, Diadema 04021-001, SP, Brazilbatista@unifesp.br (W.L.B.); 5 Laboratório de Imunologia Celular e Bioquímica de Fungos e Protozoários, Unidade José Alencar, Universidade Federal de São Paulo campus Diadema, 4° andar, Rua São Nicolau, 210, Centro, Diadema 09913-030, SP, Brazil

**Keywords:** extracellular vesicles, *Leishmania (Leishmania) amazonensis*, virulence, macrophages

## Abstract

*Leishmania* spp. is the aetiologic agent of leishmaniasis, a disease endemic in several developing countries. The parasite expresses and secretes several virulence factors that subvert the macrophage function and immune response. Extracellular vesicles (EVs) can carry molecules of the parasites that show immunomodulatory effects on macrophage activation and disease progression. In the present work, we detected a significantly higher expression of lpg3 and gp63 genes in *Leishmania amazonensis* promastigotes recovered after successive experimental infections (IVD-P) compared to those cultured for a long period (LT-P). In addition, we observed a significantly higher percentage of infection and internalized parasites in groups of macrophages infected with IVD-P. Macrophages previously treated with EVs from LT-P showed higher percentages of infection and production of inflammatory cytokines after the parasite challenge compared to the untreated ones. However, macrophages infected with parasites and treated with EVs did not reduce the parasite load. In addition, no synergistic effects were observed in the infected macrophages treated with EVs and reference drugs. In conclusion, parasites cultured for a long period in vitro and recovered from animals’ infections, differently affected the macrophage response. Furthermore, EVs produced by these parasites affected the macrophage response in the early infection of these cells.

## 1. Introduction

Leishmaniasis belongs to a neglected group of diseases caused by several protozoa species of the *Leishmania* genus, endemic in approximately 100 countries, located mainly in tropical and subtropical regions [1]. The disease has a wide spectrum of clinical manifestations, often associated with specific species or subgenera of *Leishmania* [2]. However, leishmaniasis can be clinically classified into two groups: visceral leishmaniasis (VL) and cutaneous or tegumentary leishmaniasis (CL), the most prevalent clinical form [2]. Currently, disease control is based on prophylactic measures to combat the vector, control of reservoirs (mainly domestic dogs), health education activities, adequate diagnosis, and treatment of infected individuals [3]. However, therapeutic options for treating leishmaniasis are based on drugs that have problems, such as their cost, resistance development, side effects, and toxicity, limiting their use and effectiveness [4]. Furthermore, it is common for patients not to complete the therapeutic regimen due to the toxic effects, long duration of therapy, and difficult access to the hospital environment to receive intravenous treatment [5].

*Leishmania* is a parasite with a heteroxenic life cycle that has two main life forms: promastigotes, which are present in infected female sandflies (vector) and regurgitated during the blood meal of the vector, and amastigotes, detected mainly inside the phagocytic cells of the vertebrate host [2]. *Leishmania* promastigotes interact early with a complex extracellular matrix, immune system molecules, and phagocytic tissue cells [6]. The interaction between *Leishmania* and macrophages is one of the most studied, as these cells are important to parasitic control [7]. Macrophages can eliminate phagocytosed parasites by producing microbicidal molecules (nitric oxide—NO and reactive oxygen species—ROS), and they can stimulate the effector immune response by releasing pro-inflammatory cytokines (TNF-α and IL-6, for example) [7]. Although macrophages and other immune cells have tools for controlling infection (phagocytosis or the production of microbicidal molecules NO and ROS), *Leishmania* has several virulence factors capable of subverting the effector response of macrophages [8]. The zinc-dependent metalloprotease glycoprotein 63 kDa (GP63) is a protease produced by *Leishmania* and is considered a virulence factor, as it can cleave the host molecules responsible for the response against the parasite [9]. GP63 can target several transcription factors and proteins involved in macrophage activation pathways, and the degradation of these components can prevent the complete activation of macrophages, consequently impairing the destruction of the internalized parasite [10]. GP63 cleaves complement protein C3b into an inactive form (iC3b), allowing the parasite to escape from the complement system [11]. Intracellularly, GP63 favors parasite survival by degrading the different molecules important to cell signaling and activation, modulating the macrophage’s functions [12,13,14]. Lipophosphoglycan (LPG) is another *Leishmania* virulence factor that participates in the initial infection process of macrophages. LPG delays lysosome-to-phagosome fusion and the assembly of NADPH oxidase [15]. The *lpg3* encodes a protein that belongs to an endoplasmic reticulum chaperone (HSP90) homologous to glucose-regulated protein 94 (GRP94) [16]. In *Leishmania*, the LPG3 protein is involved with LPG synthesis and the production of GPI-anchored molecules [16]. Although the *lpg3* gene is also expressed in the non-virulent *Leishmania tarentolae* [17], the protein LPG3 is immunogenic [18], modulates the host immune response [19], and is considered a virulence factor in *Leishmania* [20,21]. Aside from these, *Leishmania* produces several virulence factors that can inhibit/subvert macrophages, such as cysteine proteases, arginase, ef-1α, heat-shock proteins (HSPs), and others [14].

Currently, several studies have shown that these and other important virulence factors may be present in the extracellular vesicles (EVs) spontaneously released by several species of *Leishmania* [22,23,24,25]. EVs are a complex and heterogenous group of particles that are released into the extracellular space by prokaryotic and eukaryotic cells [26]. These particles comprise a lipid bilayer and can carry proteins, glycoproteins, lipids, nucleic acids, and secondary metabolites from one cell to another [26]. According to their origin, size, and molecular constitution, EVs can generically be classified into exosomes and ectosomes. However, each EV group can still be subclassified into size and/or markers [27]. Our group and others showed that *Leishmania* EVs co-injected with parasites participate in the progression of experimental cutaneous leishmaniasis, affecting the immune response and favoring the parasite infection [23,25]. However, BALB/c mice immunized with EVs released by *L. amazonensis* promastigotes with different virulence profiles modulated the immune response and were partially protected from the progression of infection [28]. Given that virulence factors can contribute to the subversion of the macrophage response and that EVs can contain different parasite virulence factors, gaining a better understanding of how EVs obtained from parasites with different degrees of virulence modulate macrophage activation can help to better understand the role of EVs in the host–parasite relationship and the subversion of macrophage functions.

In this work, we improved the characterization of the parasites obtained after a successive infection in animals (IVD-P) and cultured them in vitro for a long-term duration (LT-P). In addition, we evaluated the EVs released by these parasites, their influence on macrophage infection (before and after *L. amazonensis* infection), and cytokine production. In vitro experiments were also carried out to assess the potential use of EVs in the treatment of cutaneous leishmaniasis.

## 2. Materials and Methods

### 2.1. Parasites

The MHOM/BR/1973/M2269 strain of *L. amazonensis* was used in this study. Long-term parasites (LT-P) and in vivo-derived parasites (IVD-P) were obtained as previously described [28]. The parasites (promastigotes forms) were cultured at 26 °C in 199 medium (Gibco, Life Technologies, Grand Island, NY, USA) supplemented with 4.2 mM sodium bicarbonate, 4.2 mM HEPES, 1 mM adenine (Sigma, St. Louis, MO, USA), 5 µg/mL of hemin (bovine type I) (Sigma), and 10% fetal bovine serum (FBS) (Gibco). The cultures were maintained until the parasites reached stationary growth.

### 2.2. RNA Extraction

*L. amazonensis* promastigotes, with distinct virulence profiles (LT-P and IVD-P), were subjected to RNA extraction using the TRIzol^®^ reagent, following the manufacturer’s recommendations. RNAs were quantified in a spectrophotometer (Nanodrop 2000c, Thermo Fisher, Scientific Brand, Grand Island, NY, USA), and the samples with 260/280 nm and 260/230 nm ratios between 1.8–2.0 were analyzed to integrity using 1.5% agarose gel. The RNA samples were stored at −80 °C to avoid degradation.

### 2.3. Quantitative Reverse Transcriptase-Polymerase Chain Reaction

A quantitative reverse transcriptase-polymerase chain reaction (qRT-PCR) was employed to analyze the expression of the RNAs extracted from the LT-P and IVD-P. The recommendations of the *Minimum Information for Publication of Quantitative Real-time PCR Experiments* (MIQE) guidelines [29] were followed to guarantee the quality of the results. First, samples with a high integrity and quality of 260/280 and 260/2030 ratios were treated with DNAse (RQ1 RNase-free DNase; Promega, Madison, WI, USA) to avoid contamination with genomic DNA. Then, the cDNA synthesis was performed with the ProtoScript M-MuL V First Strand cDNA Synthesis Kit (New England Biolabs Inc., Ipswich, MA, USA). Treatment with DNAse and the cDNA preparation followed the manufacturer’s instructions.

The real-time PCR reactions were performed using the Sybr Green reagent (Applied Biosystems, Thermo Fisher Scientific). The reverse transcription reaction was performed using 1 μL of cDNA, 5.0 μL of SYBR Green Master Mix (Thermo Fisher), and 2.0 μL of each oligonucleotide (1.0 μM). Real-time PCR reactions were performed in StepOne Plus equipment (Applied Biosystems), and the cycle parameters were as follows: 10 min at 50 °C for enzyme activation, denaturation for 5 min at 95 °C, and 40 cycles of 95 °C for 30 sec and 60 °C for 1 min. Internal negative controls were performed using the cDNA prepared with RNA samples without adding the transcriptase reverse for the cDNA preparations and using the reaction mix (SYBR Green Master Mix and each oligonucleotide) without the cDNA template (no template control–NTC). In both cases, no amplifications were detected.

The following target genes were used: arginase (*arg*), glycoprotein 63 (*gp63*), heat-shock protein 78 (*hsp78*), lipophosphoglycan 3 (*lpg3*), elongation factor 1-alpha (*ef-1α*), oligopeptidase B (*oligo b*), cysteine protease b (*cpb*), and cysteine protease c (*cpc*). In addition, α-tubulin (*α-tub*) was used as a housekeeping gene. All sequences are described in Supplementary Table S1. The reaction quality was based on the dissociation curves, and the results were analyzed using the StepOne software program (Applied Biosystems). To calculate the relative gene expression, the baseline was adjusted to two or three cycles before the detection of the fluorescent signal, and the cycle threshold (CT) was determined (CT is the number in which the cycle reached the fluorescence threshold line—the value above the background). The evaluated CT was always in the exponential phase of the amplification to increase the accuracy of the measurement of transcripts in the sample. The CT values of the target genes and the α-tub were used to calculate the ΔCT. The 2^−ΔΔCT^ is the value representing the relative expression of the analyzed gene.

Before the gene expression assays, the efficiencies of all primers were evaluated by constructing standard curves with four or five different points of the dilution using the reference samples. The CT values of each dilution point were used to build the standard curve and to calculate the efficiency of the primers using the equation E = 10^(−1/slope)^ − 1 (E corresponds to the efficiency, and slope is the slope of the standard curve). Then, the efficiencies of the primers of target genes and α-tub were compared.

All experiments were performed in triplicate with at least three biological samples. Differences in the relative expression levels of the target genes (fold change) were determined by comparing the LT-P as the reference samples (control) with IVD-P. The gene expression for the reference sample was adjusted to 1.

### 2.4. Phagocytosis Assay

The murine macrophage lineage, RAW 264.7, was cultured in Roswell Park Memorial Institute (RPMI) 1640 medium (ThermoFisher), supplemented with 2 mM L-glutamine, 25 mM 4-(2-hydroxyethyl)-1-piperazineethanesulfonic acid (HEPES), with 10% FBS added (Gibco). The cultures were maintained in a humid atmosphere with 5% CO_2_ up to 90% confluence. Then, 1 × 10^5^ cells/well were seeded in 24-well plates. Macrophage cultures were infected with *L. amazonensis* LT-P or IVD-P promastigotes in the stationary phase, suspended in RPMI 1640 medium plus 10% FBS at a ratio of 10 parasites to 1 cell (multiplicity of infection-MOI 10:1). After 3 h of contact, non-internalized parasites were removed by washing with serum-free RPMI medium and the cultures were incubated for 24 h in a humidified atmosphere at 33 °C in 5% CO_2_. Then, the supernatants were removed, the cells stained with hematoxylin and eosin (Panotic Kit), and the percentage of infection and the number of internalized parasites were evaluated by observation under a light microscope. Each experimental group was performed in triplicate, and 100 cells were counted per slide in random fields.

### 2.5. Obtaining Extracellular Vesicles of L. amazonensis Promastigotes

The parasites were cultured as promastigotes until the stationary phase. Then, EVs were obtained as described by [25]. Briefly, 10^8^ parasites/mL were added to 1.5 mL microtubes containing RPMI medium with 2% glucose added and kept at 26 °C for 4 h. Afterward, the microtubes were centrifuged to separate the parasite from the supernatant containing the EVs. Next, the supernatant was subjected to several cycles of centrifugation and ultracentrifugation: 500× *g* for 10 min at 4 °C, 1500× *g* for 10 min at 4 °C, and 10,000× *g* for 10 min at 4 °C to remove cellular debris. Then, the supernatant was ultracentrifuged at 100,000× *g* for 1 h [30]. Finally, the pellet that formed was diluted in phosphate-buffered saline (PBS) and centrifuged at 100,000× *g* for 1 h. The pellet contained EVs that were diluted in sterile, filtered PBS and stored at −20 °C.

EVs were analyzed by nanoparticle tracking analysis (NTA) and flow cytometry. NTA was performed using Nanosight NS300 equipment (Malvern Instruments Ltd., Malvern, United Kingdom). First, approximately 1 mL of PBS was injected into the equipment to eliminate interferences, and then 1 mL of each sample, diluted 10- to 100-fold in sterile PBS, was analyzed. Data were collected under the following conditions: images were captured in triplicate, 30 s/capture, the camera level was set to 14, and we used the same threshold for all analyses. NTA software, version 2.3 build 0017, was used to analyze all images. EVs were also analyzed by flow cytometry. Briefly, unstained EVs were resuspended in 400 μL of washing buffer, and the samples were acquired on a Symphony A1 flow cytometer (BD Bioscience, San Jose, CA, USA) and FACSDiva software (BD). Then, megamix beads (BD), with sizes ranging from 160 to 500 nm, were used as a parameter to evaluate the profile size in the EVs released by LT-P and IVD-P parasites. All data were collected on the small particle scatter detector for the scatter channel (SP SSC). Acquired data were analyzed using FlowJo software (FlowJo, FlowJo™ Software for Mac, Ashland, OR, USA).

### 2.6. L. amazonensis EVs Labeling with PKH26

*L. amazonensis* EVs from LT-P and IVD-P parasites were stained with the cell membrane fluorescent marker PKH26 (Sigma), according to the manufacturer’s instructions. Briefly, 4 × 10^7^ EVs of each sample were diluted in 1 mL of PBS. Then, 2 µL of the cell membrane fluorescent marker PKH26 was diluted in 498 µL of Reagent C and added to each sample. After incubation for 30 min at 37 °C, 500 µL of sterile PBS was added. The samples were ultracentrifuged for 16 h, the supernatant was discarded, and the pellet was resuspended in 500 µL of RPMI medium plus 10% FBS (the FBS was previously ultracentrifuged for 8 h to remove FBS-EVs). The same procedure was performed using only PBS in the absence of *L. amazonensis* EVs (negative control). Parasites stained with PKH26 were used as the positive control.

### 2.7. Uptake of EVs by Macrophages

RAW 264.7 cell cultures were seeded in 24-well plates at a density of 1 × 10^5^/well. After 2 h, the cells were treated with a ratio of 100 EVs stained with PKH26 per macrophage. The negative control was given as macrophages without EVs, and the second control was given as macrophages incubated with EVs not labeled with PKH26. The cultures were incubated for 24 h at 37 °C in a 5% CO_2_ atmosphere. After incubation, the supernatants were removed, and 300 µL of 1 mM ethylenediaminetetraacetic acid (EDTA) was added to each well to detach the cells. After 1 min at room temperature, the wells were scraped, and their contents were transferred to 1.5 mL microtubes. The samples were centrifuged for 5 min at 1200× *g*. The supernatant was removed, and the pellet was resuspended in 500 µL of sterile PBS for flow cytometry or confocal microscopy analyses.

The FACSCalibur flow cytometer (BD) was used to acquire and analyze the cell percentages labeled with EVs stained with PKH26. The CellQuest software was used for data acquisition, and statistical analyses were performed using the FlowJo software.

The microscopy images were obtained using a confocal microscope (SP8 Lightning, Leica Microsystems). For this proposal, RAW 264.7, treated with PKH26-labeled EVs, was fixed with cold methanol for 1 min, permeabilized with 0.05% Triton X-100 for 10 min at room temperature (RT), and additionally stained with 300 nM 4′,6′-diamino-2-phenyl-indole (DAPI) for 10 min at RT. Then, the macrophages were distributed into wells of adapted slides for analysis under a confocal microscope. The images were analyzed using the ImageJ analysis software (version 1.53i) through corrected total cell fluorescence (CTCF).

### 2.8. Cytokine Production

The cytokine production by macrophages was evaluated using a cytometric beads array (Mouse Inflammation kit-CBA, BD Bioscience). The supernatants of macrophage cultures were used to evaluate the presence of the inflammatory cytokines. Reactions were carried out according to the manufacturer’s instructions, and samples were acquired in a FACSCalibur (BD) flow cytometer. Cytokine analysis was performed by calculating the standard curve for each cytokine. Data were expressed in pg/mL. All analyses were performed using the FCAPArray (BD) software.

### 2.9. NO Production

The NO production was analyzed after the incubation of macrophages with EVs from LT-P or IVD-P parasites for 24 h at 37 °C in a 5% CO_2_ atmosphere. The wells were washed with PBS, and the fluorescent reactive species marker 4,5-diaminofluorescein diacetate (DAF 2-DA) (Sigma) was added at a final concentration of 5 µM. The plate was protected from light and incubated at 37 °C with 5% CO_2_ for 30 min. The wells were washed with PBS, and 300 µL of RPMI was distributed per well. The plate was protected from light and incubated at 37 °C with 5% CO_2_ for 30 min. The wells were washed with PBS, 300 µL of EDTA 1 mM was added per well, and the plate was incubated for 5 min. After this period, the adhered cells were detached and centrifuged at 2000× *g* for 5 min at 4 °C. The supernatants were discarded, and the pellet was resuspended in 500 µL of PBS for analysis on the FACSCalibur flow cytometer.

### 2.10. Stimulation of Macrophages for Cytokine Production via L. amazonensis Infection after EVs Internalization

**Figure 1:**
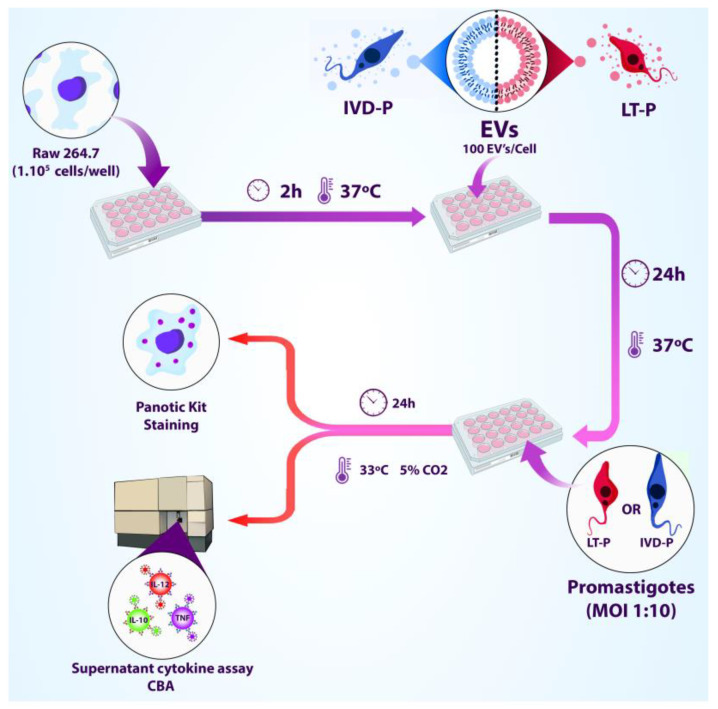
Workflow of treating RAW 264.7 macrophage lineage with EVs released by LT-P or IVD-P before macrophage infection with *L. amazonensis* (adapted from BioRender, https://www.biorender.com/, accessed on 3 November 2023).

The RAW 264.7 cells were infected after treatment with EVs. Thus, the macrophages were treated with EVs (100 particles/cell) for 24 h, following the same conditions described above. Then, the macrophages were infected with the correlate parasites MOI 10:1 (LT-P or IVD-P), and the cultures were maintained for 24 h in RPMI medium with 10% FBS added in a humidified atmosphere at 33 °C in 5% CO_2_. The supernatants were collected, and the cytokine production was evaluated by CBA. Cells were stained to determine the percentage of infection and the number of internalized parasites (Figure 1). The following controls were used: (1) macrophages untreated and uninfected; (2) macrophages untreated and infected with the parasites; (3) macrophages stimulated with LPS. Each experimental group was performed in triplicate.

### 2.11. Identification of EVs’ Activity in the Presence of Anti-Leishmania Drugs in Promastigote-Bearing Macrophages

**Figure 2:**
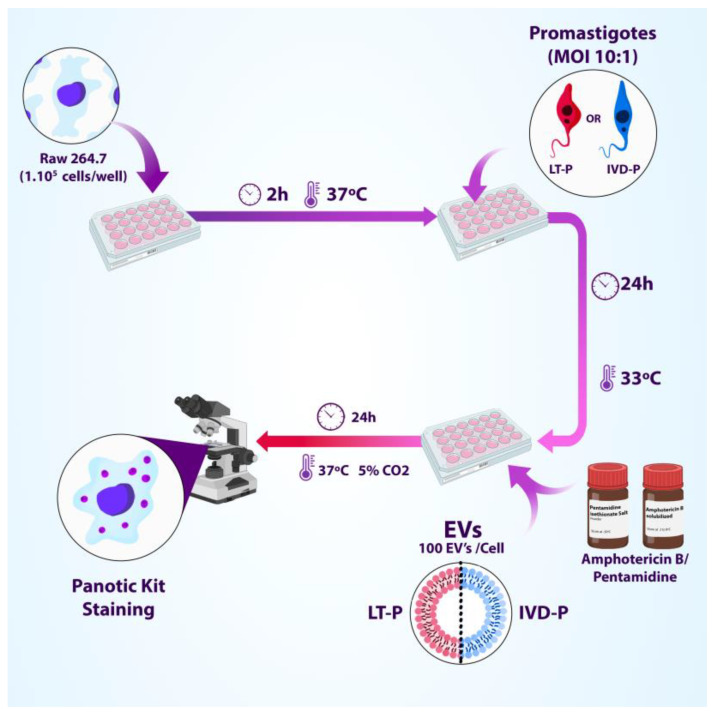
Experimental design of macrophage infection (RAW 264.7) with LT-P or IVD-P followed by subsequent treatment with different concentrations of Amphotericin b or Pentamidine in the presence or absence of EVs (adapted from BioRender, https://www.biorender.com/, accessed on 3 November 2023).

To assess the effect of the EVs in the treatment with the reference drugs for leishmaniasis, macrophages were infected with LT-P or IVD-P promastigotes for 24 h. Then, cultures were treated with 3 concentrations of Amphotericin b (Sigma) or Pentamidine (Sigma) in the presence or absence of EVs from LT-P or IVD-P (100 EVs/macrophage). After 24 h, the culture supernatants were removed, and the cells were stained to assess the percentage of infection and the number of internalized parasites (Figure 2).

### 2.12. Statistical Analysis

Statistical analyses were performed using analysis of variance (ANOVA) followed by the Tukey test using GraphPad Prism 3.0, version 8.3.0 for Windows (GraphPad Software, La Jolla, CA, USA). In addition, the Student’s *t*-test was used to determine the statistical significance between the two groups. All experiments were performed at least twice with triplicate groups.

## 3. Results

### 3.1. After Animal Infection, L. amazonensis Showed an Increase in lpg3 and gp63 Expression and Higher Infection in Macrophages

**Figure 3:**
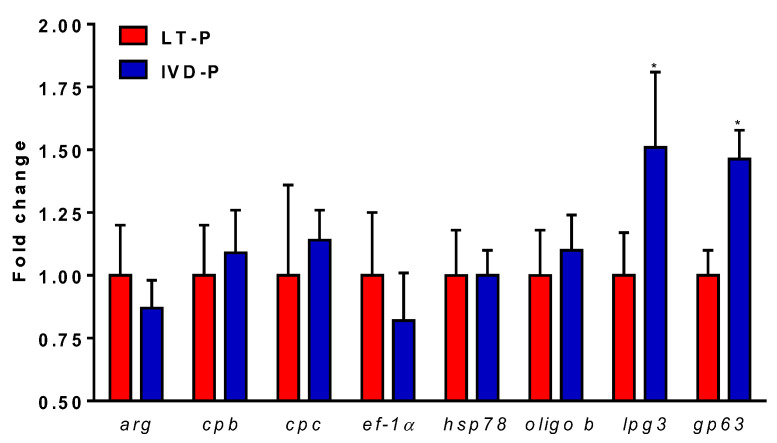
Relative expression analyses of virulence genes expressed in *L. amazonensis* promastigotes of LT-P and IVD-P. The bars represent the mean of the triplicates, and the error bars are the standard deviation. Student’s *t*-test comparing LT-P and IVD-P, * *p* < 0.05. The graph is representative of two independent experiments.

A real-time PCR (qRT-PCR) was performed to evaluate the differential gene expressions of eight classic virulence factors in *L. amazonensis* submitted to three consecutive animal infections (IVD-P). The genes evaluated were selected according to the literature and data from our group [14,25,28]. Our results showed that IVD-P has a significantly higher expression of *lpg3* and *gp63* genes compared to the *L. amazonensis* cultured for long-term in vitro LT-P (Figure 3). No statistical differences were observed in the gene expressions of *arg*, *cpb*, *cpc*, *hsp78*, *ef1-α*, and *oligo-b* genes when comparing LT-P and IVD-P (Figure 3).

**Figure 4:**
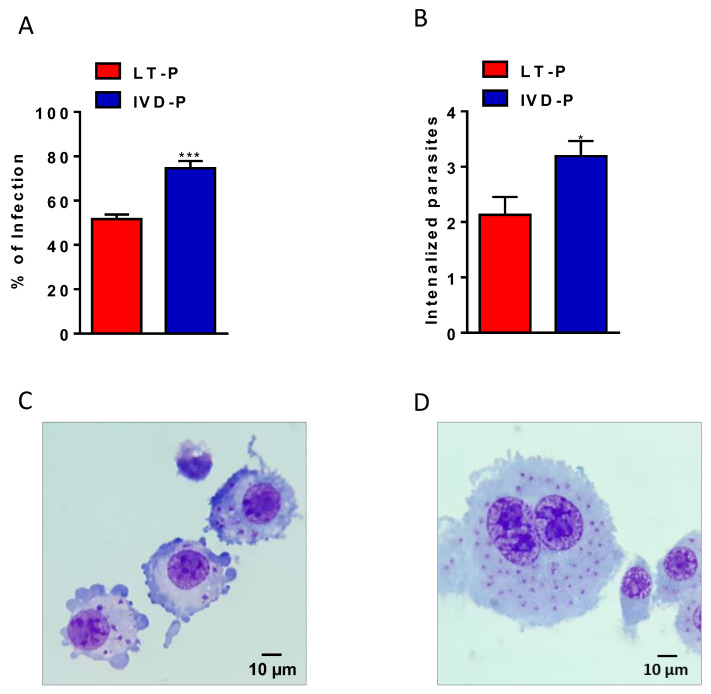
Phagocytosis assay of *L. amazonensis* promastigotes with different virulence profiles. RAW 264.7 cell lineage infected with LT-P or IVD-P promastigotes. (**A**) Percentage of macrophage infection; (**B**) average of internalized parasites; (**C**) representative microphotograph of macrophage infection with LT-P; (**D**) representative image of macrophage infected with IVD-P, both 1,000× magnification. Each group was performed in triplicate. The bars represent the mean of the triplicates, and the error bars are the standard deviation. Student’s *t*-test * *p* < 0.05 and *** *p* < 0.001. Representative data of 3 independent experiments.

In addition, the ability to infect macrophages was evaluated in the parasites recovered after animal infection (IVD-P) and cultured in vitro for a long-term duration (LT-P). The phagocytosis assays showed a significantly higher percentage of infection (Figure 4A) and internalized parasites (Figure 4B) in the macrophages infected with IVD-P compared to those infected with LT-P. Figure 4C,D are representative images of the RAW 264.7 macrophages infected with *L. amazonensis* LT-P and IVD-P, respectively.

### 3.2. Morphological and Functional Analyses of EVs Released by L. amazonensis Promastigotes before and after Animal Infection

**Figure 5:**
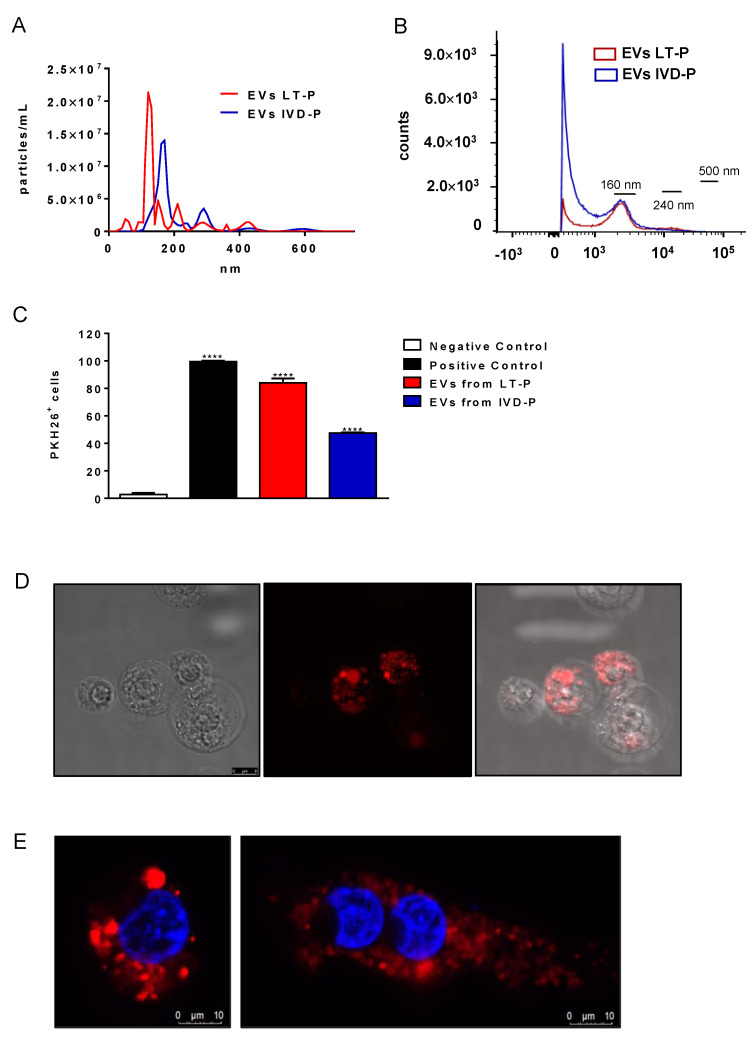
EVs released by *L. amazonensis* promastigotes with different virulence profiles (IVD-P and LT-P). (**A**) Size profile and concentration (particles/mL) of EVs analyzed by NTA. (**B**) Flow cytometry acquisition of non-stained EVs from LT-P and IVD-P parasites. (**C**) Flow cytometry of RAW 264.7 cells after uptake of *L. amazonensis* EVs stained with PKH26. Bars represent the mean percentage of label cells after 3 measurements, and error bars show the SD. ANOVA following Tukey post-test. **** *p* < 0.0001. Negative control-unlabeled cells; positive control cells labeled with PKH26. (**D**) Representative image of macrophage cells after uptake of *L. amazonensis* LT-P EVs stained with PKH26 (red marker). The uptake experiments were performed twice with similar results. (**E**) Representative images of macrophage cells after uptake of *L. amazonensis* LT-P EVs (left image) and IVD-P EVs (right image) stained with PKH26 (red marker). The cell nucleus was labeled with DAPI (blue marker).

EVs produced by LT-P and IVD-P were evaluated by NTA analysis for their size and concentration (Figure 5A). No significant differences were observed when comparing the size or concentration of EVs released by LT-P and IVD-P. In addition, EV size profiles were also characterized using flow cytometry (Figure 5B). Similar to the results obtained by the NTA, there were no significant differences in the size profile when comparing the EVs released by LT-P and IVD-P (Figure 5B).

The uptake of EVs by macrophages was analyzed by flow cytometry and confocal microscopy. First, the EVs from LT-P and IVD-P were labeled with the fluorescent marker PKH26. Figure 5C shows a significant percentage of cells positive to PKH26 in the groups incubated with stained EVs, compared to the negative control (**** *p* < 0.0001). The group treated with LT-P Evs showed a significantly higher percentage of PKH26-positive cells than those treated with IVD-P (**** *p* < 0.0001). Figure 5D,E and Supplementary Figure S1 show, by confocal microscopy, the presence of fluorescent marker PKH26 inside the RAW 264.7 exposed to stained EVs, suggesting the incorporation of these particles by cells.

**Figure 6:**
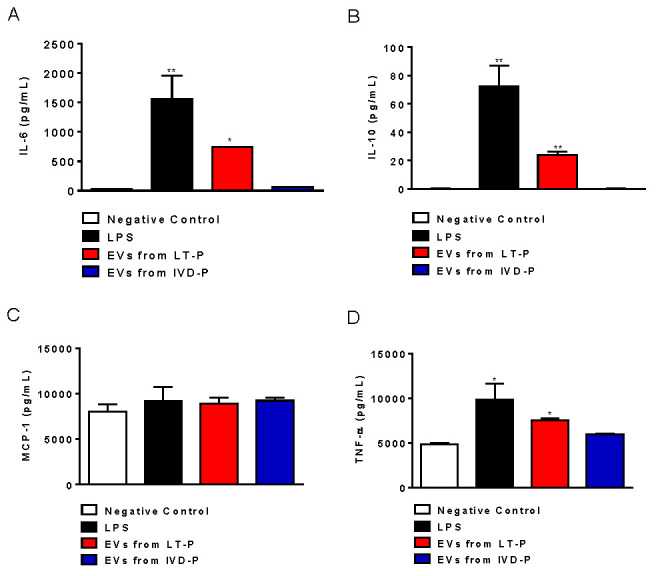

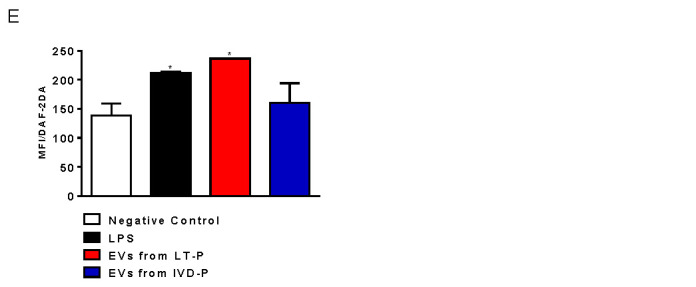
Cytokine and NO production in RAW 264.7 cells treated with EVs from LT-P or IVD-P. The control received LPS. (**A**) Measurements of IL-6, (**B**) IL-10, (**C**) MCP-1, and (**D**) TNF-α cytokine levels in cell supernatants. (**E**) Intracellular NO production in RAW 264.7 cells after stimulation with EVs from IVD-P or LT-P. Data acquisition was performed using a FACSCalibur cytometer, and subsequent analyses were performed using the FlowJo software. The graph represents the median fluorescence intensity (MFI) for the DAF-2DA probe. The bars indicate the mean, and the error bars denote the standard deviation of the groups. Statistics were performed using ANOVA, followed by the Tukey post-test (* *p* < 0.05,  ** *p* < 0.01). The graphs are representative of two independent experiments.

The macrophages stimulated with LT-P EVs showed a significant increase in IL-6, IL-10, and TNF-α production compared to the control (Figure 6A–D, respectively). EVs from IVD-P did not induce significant production of any of the cytokines analyzed (Figure 6A–D). The cytokines IFN-γ and IL-12p70 were not detected in the culture supernatants. Similar effects were observed in the NO production. There was a significant increase in the fluorescence intensity after adding DAF-2DA (the NO fluorescence probe) in the cells stimulated with LT-P EVs, compared to the control. No significant increase in NO production was observed in the cells stimulated with EVs from IVD-P (Figure 6E).

### 3.3. The Macrophage Infection and Cytokine Production after Treatment with EVs Are Influenced by Virulence

**Figure 7:**
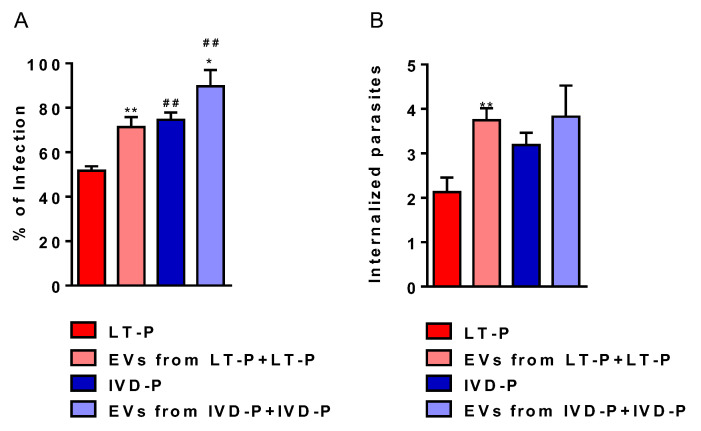
Phagocytosis assay of *L. amazonensis* promastigotes with different virulence profiles in the presence of the respective EVs. RAW 264.7 cells were previously stimulated with the EVs released by LT-P or IVD-P promastigotes. After 24 h, macrophages were infected with correlated parasites LT-P or IVD-P (EVs from LT-P or IVD-P + LT-P or IVD-P infection). (**A**) Percentage of infection; (**B**) average of internalized parasites. The bars represent the mean of the triplicates, and the error bars are the standard deviation. Statistics were performed using ANOVA, followed by the Tukey post-test. * *p* < 0.05,  ** *p* < 0.01, compared to groups not treated and treated with EVs (LT-P compared to EVs from LT-P + LT-P infection or IVD-P compared to EVs from IVD-P + IVD-P infection). ## *p* < 0.01, comparison between groups not treated with EVs (LT-P and IVD-P) or treated with EVs (EVs from LT-P + LT-P infection and IVD-P + IVD-P-P infection). The graphs are representative of two independent experiments.

Previous stimulation with EVs led to a significant increase in macrophage infection (Figure 7A) and the number of internalized parasites in the groups treated with LT-P EVs (Figure 7B). On the other hand, there was no change in the number of internalized parasites in the groups treated with IVD-P EVs (Figure 7B).

**Figure 8:**
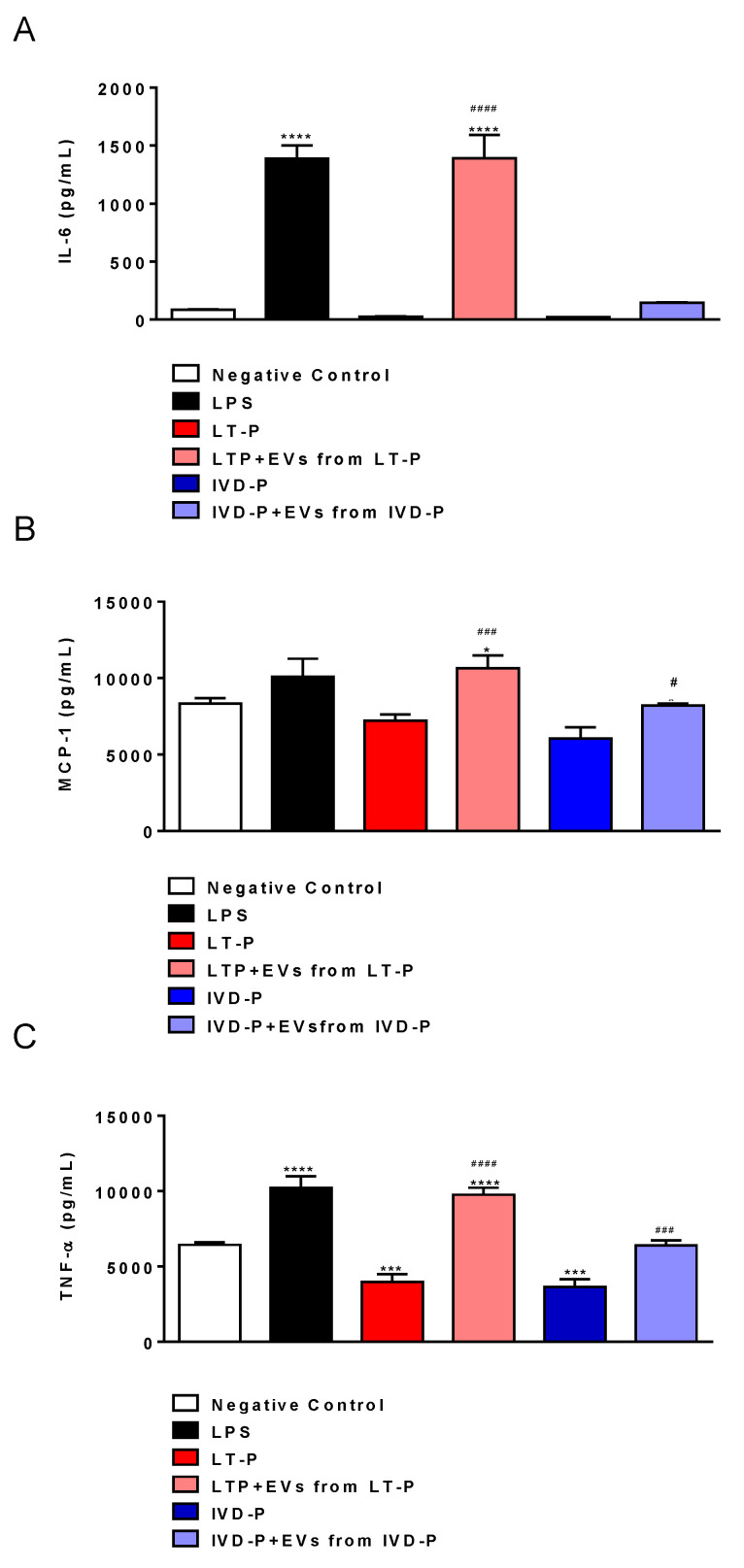
Cytokine production by RAW264.7 cells infected with LT-P or IVD-P promastigotes previously treated with the related EVs. The negative control was non-infected and had no treated cells. The positive control was stimulation with LPS. Supernatants were collected after 24 hours of infection. (**A**) Measurements of IL-6, (**B**) MCP-1, (**C**), and TNF-α cytokine levels in cell supernatants. The bars indicate that the mean and the error bars denote the standard deviation of the groups. Statistics were performed using ANOVA, followed by the Tukey post-test. (*) indicates a significant difference in relation to the negative control group (* *p* < 0.05,  *** *p* < 0.001,  and **** *p* < 0.0001). (^#^) represents a comparison between the groups infected with the parasites without pre-treatment with EVs and groups treated with EVs and subsequently infected with the parasites (^#^ *p* < 0.05,  ^###^ *p* < 0.001,  and ^####^ *p* < 0.0001).

The cytokines IL-6, TNF-α, and MCP-1 showed significantly higher levels in the groups treated with LT-P EVs before the infection with LT-P compared to the untreated and uninfected groups (Figure 8A–C). The macrophages previously treated with EVs from the parasites recovered from animal infection (EVs from IVD-P) and infected with the cognate parasite (IVD-P) were unable to increase the production of pro- or anti-inflammatory cytokines (Figure 8A–C). The cytokines IL-10, IFN-γ, and IL-12p70 were not detected in any of the groups evaluated.

### 3.4. EVs Had No Synergistic Effect on RAW 264.7 Macrophages Infected with L. amazonensis Treated with Pentamidine or Amphotericin b

**Figure 9:**
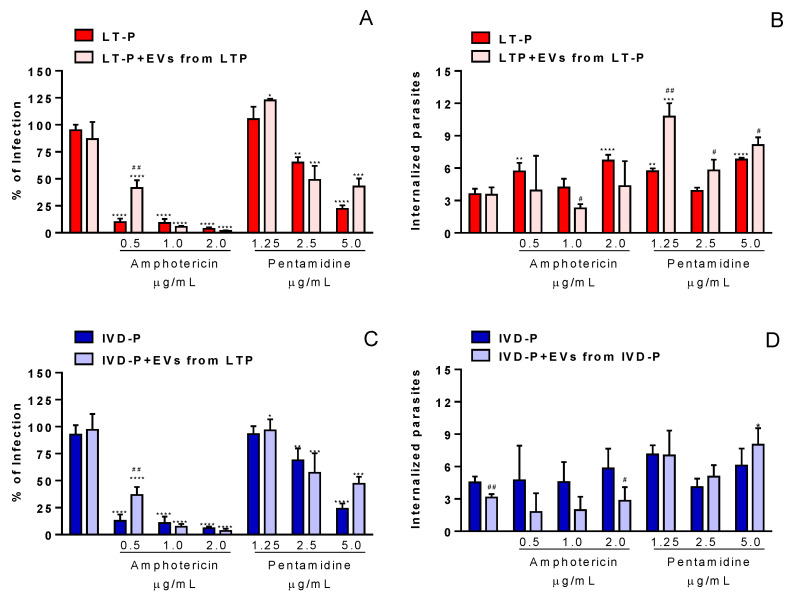
Percentages of macrophage infection and the number of internalized parasites in RAW 264.7 cells infected with *L. amazonensis* and treated with 3 concentrations of Amphotericin b or Pentamidine in the presence or absence of EVs. (**A**,**B**) Infection with LT-P and treatment with Amphotericin b or Pentamidine and LT-P EVs, and (**C**,**D**) infected with IVD-P and treated with Amphotericin b or Pentamidine and IVD-P EVs. The bars represent the mean of the triplicates, and the error bars are the standard deviation. Two-way ANOVA, followed by Tukey test. * *p* < 0.05, ** *p* < 0.01; *** *p* < 0.001; **** *p* < 0.0001 compared to the control group (infected or infected and treated with the respective EV); ^#^ *p* < 0.05, ^##^ *p* < 0.01 compared to group infected and treated with the same drug concentration in the absence of EVs. The experiment was performed twice with similar results.

The influence of EVs after an established infection in macrophages was also evaluated. This analysis was performed by treating cells with EVs in the presence or absence of the reference drugs. There were no changes in the percentage of infected cells and the number of internalized parasites in the groups treated with EVs by LT-P alone (Figure 9A,B). However, at a concentration of 0.5 µg/mL of Amphotericin b and 5.0 µg/mL of Pentamidine, EVs increased the percentage of infection in the macrophages infected with LT-P (Figure 9A). Considering the number of internalized parasites, it was observed that the presence of LT-P EVs significantly reversed the effect of Pentamidine in the groups infected with LT-P parasites, leading to an increase in the number of internalized parasites in the groups treated with those EVs (Figure 9B).

In the macrophage groups infected with IVD-P and treated with IVD-P EVs, it was observed that the percentage of infection had similar results to those observed in the groups infected with IVD-P and treated with IVD-P EVs (Figure 9C). Macrophages infected with IVD-P and treated with 0.5 µg/mL of Amphotericin b in the presence of EVs from IVD-P increased the percentage of infection compared with the groups not treated with EVs (Figure 9C). A similar effect was observed when cells were treated with 5.0 µg/mL of Pentamidine in the presence of IVD-P EVs (Figure 9C). Considering the number of internalized parasites, a significant reduction was observed in the group treated only with IVD-P EVs and in the group treated with 2.0 ug/mL of Amphotericin b (Figure 9D). Regarding treatment with Pentamidine, an increase in internalized parasites was observed in the group treated with IVD-P EVs and 5.0 ug/mL of the drug.

## 4. Discussion

Several studies have shown that the long-term culture of *L. infantum*, *L. major*, and *L. amazonensis* alters the expression of virulence factors, changes the parasite metabolism, and impairs their ability to infect mice [28,31,32,33,34]. Although these studies are still in their infancy, they shed light on the importance of evaluating the loss of infectivity of *Leishmania* after a long period of culture when in vitro.

In this work, we showed that after successive passages in animals, *L. amazonensis* promastigotes (IVD-P) increased the expression of *lpg3* and *gp63* (two important virulence factors), as compared to the parasites submitted to long-term in vitro culturing (LT-P). In addition, the macrophages infected with IVD-P showed a higher percentage of infection and number of parasites internalized compared to the cells infected with LT-P. Previous studies from our group showed that EVs from *L. infantum* BA262 LPG-deficient mutant (1lpg1) (LPG-KO) did not affect the NO and cytokine productions of murine macrophages. However, like EVs from the present work, EVs from *L. amazonensis* (Ba125 strain) were also very pro-inflammatory for murine macrophages via TLR4/TLR2 [35]. Furthermore, a prolonged expression of LPG by *Leishmania* after infection of some cell types can have a role in the intracellular survival of the parasite [36]. Thus, it is plausible to suppose that the higher expression of the *lpg3* gene observed in IVD-P promastigotes is related to the increased number of parasites observed in infected macrophages.

Aside from *lpg3*, IVD-P also showed an increase in *gp63* expression, a metalloprotease with pleiotropic functions in a *Leishmania* infection [14]. IVD-P promastigotes infected macrophages more efficiently than LT-P promastigotes, suggesting that virulence factors can contribute to IVD-P infectivity. This hypothesis is highly speculative; however, studies on parasites that lost one of these two virulence factors showed a decrease in their ability to infect macrophages and mice [37,38,39]. In addition, [34] showed that the *L. amazonensis* cultured for a long-term duration was less infective in vitro compared to the parasites recently recovered from an in vivo infection. Therefore, it is important to highlight the importance of carrying out studies with virulent parasites since their passage for long periods in culture can alter the phenotypic and infective characteristics of the parasites.

EVs have played an important role in the *Leishmania*–host relationship, but several issues remain unclear. Given that virulence factors can also be present in the EVs released by *Leishmania* [25,28,40], we investigated the influence of EVs in early macrophage infection. First, we demonstrated that macrophages uptake EVs from LT-P and IVD-P parasites. However, RAW 264.7 cells treated with LT-P EVs showed higher labeling percentages than those treated with IVD-P EVs, suggesting that LT-P EVs may be more easily uptaken by macrophages. In addition, macrophages produced higher levels of TNF-α, IL-6, IL-10, and NO after stimulation with LT-P EVs compared to the treatment with IVD-P EVs. Thus, our data suggest that EVs released by *L. amazonensis* with a higher virulence profile can subvert the macrophage response and impair the production of inflammatory cytokines. In fact, a study on EVs from the *L. donovani* HSP100 KO parasite showed differences in cytokine production by macrophages compared to cells stimulated with EVs from wild-type parasites. A recent study showed that EVs from *L. amazonensis* expressing different levels of *gp63* (high or low expression) induce TNF-α expression in immortalized bone marrow macrophages [40]. Thus, the protein composition of EVs can influence the response of macrophages. We hypothesize that EVs released by *L. amazonensis* with different virulence profiles may have qualitatively and/or quantitatively different compositions, which can influence the macrophage response (phagocytosis and cytokine production). Thus, after consecutive infections in animals, *L. amazonensis* increases the expression of some virulence factors and improves its capacity to infect macrophages.

A second step was to evaluate the infection by *L. amazonensis* in macrophages previously treated with EVs. Our data showed that pre-treatment with EVs led to increased susceptibility to parasite infection, suggesting that EVs could have a role in the permissiveness of the macrophages to infection. On the other hand, high levels of inflammatory cytokines were maintained in the cells treated with LT-P EVs and infected with the correspondent parasite, suggesting that LT-P EVs have a strong capacity to induce an inflammatory profile. Although the exact differences between the cargo of LT-P and IVD-P vesicles have not been characterized, our laboratory showed differences in the presence of GP63 and LPG in these EVs [28]. Furthermore, in an animal immunization model, vesicles from LT-P showed a more significant protective effect and a higher IFN-γ production compared to animals immunized with IVD-P EVs [28]. The ability of LT-P EVs to induce a strong, early inflammatory response in macrophages may partially have contributed to their protective effect in immunized animals, suggesting a possible application of these EVs in developing a vaccine for cutaneous leishmaniasis.

Considering that LT-P EVs stimulated an inflammatory profile in macrophages, we evaluated the possibility of using these EVs to treat macrophages infected with *L. amazonensis*. EVs carry several bioactive molecules capable of interacting with different cell types and can alter their functionality, bringing their potential application in treating specific diseases [41]. There are several examples of EVs derived from mesenchymal cells that have been evaluated for cancer treatment and other non-chronic diseases [42,43]. In parasites, the potential application of *Fasciola hepatica* EVs for treating colitis [44] and EVs released by dendritic cells to treat *Trypanosoma cruzi* was demonstrated [45]. Our work showed that macrophages infected with *L. amazonensis* and subsequently treated with EVs did not alter the parasite load inside macrophages. Furthermore, treating infected macrophages with both EVs and reference drugs also did not show a reduction in the parasite load. Interestingly, at some concentrations of the drug, a decrease in their anti-*Leishmania* effect was observed, with an increase in the parasite load demonstrated compared to cells treated in the absence of EVs. These data corroborate our previous results that the co-injection of *L. amazonensis* EVs and parasites led to an increase in parasite load and modulated the immune response to a Th2 profile [25]. Although cells infected with the parasite and subsequently treated with EVs did not change the parasite load, using EVs to treat animals with an established infection (lesions) is still a point to be studied. In an in vivo infection, the role of EVs can be different and can contribute to a favorable outcome since cells and molecules of the immune system can interact in a complex way, leading to different activations of the response. This point should be addressed in future studies.

## 5. Conclusions

In conclusion, this study showed that *L. amazonensis* with different virulence profiles expressed *lpg3* and *gp63* differently. In addition, these parasites showed different abilities to infect macrophages. EVs released by parasites improved their ability to infect macrophages, and EVs produced by attenuated parasites (LT-P) stimulated the production of inflammatory cytokines by macrophages. However, there were no synergisms between parasite EVs and reference drugs to eliminate the parasites previously internalized by macrophages.

## Data Availability

Data are available on request from the corresponding author.

## Supplementary Materials

The following supporting information can be downloaded at: https://www.mdpi.com/article/10.3390/microorganisms11122973/s1, Table S1: Sequences of primers used for qRT-PCR reaction. Figure S1. Representative image of macrophage cells after uptake of L. amazonensis EVs stained with PKH26 (red marker). The uptake experiments were performed twice with similar results. (A) Phase contrast, and (B) fluorescence microscopy images demonstrating the uptake of labeled EVs.
