# Supplementary material for: Extracellular Vesicles Released by Leishmania (Leishmania) amazonensis Promastigotes with Distinct Virulence Profile Differently Modulate the Macrophage Functions
Source: Microorganisms. 2023 Dec 13;11(12):2973. doi: 10.3390/microorganisms11122973 (PMC10746037; doi:10.3390/microorganisms11122973)
Supplement: Supplementary file 1 [file microorganisms-11-02973-s001.zip › microorganisms-2728098-supplementary.pdf]

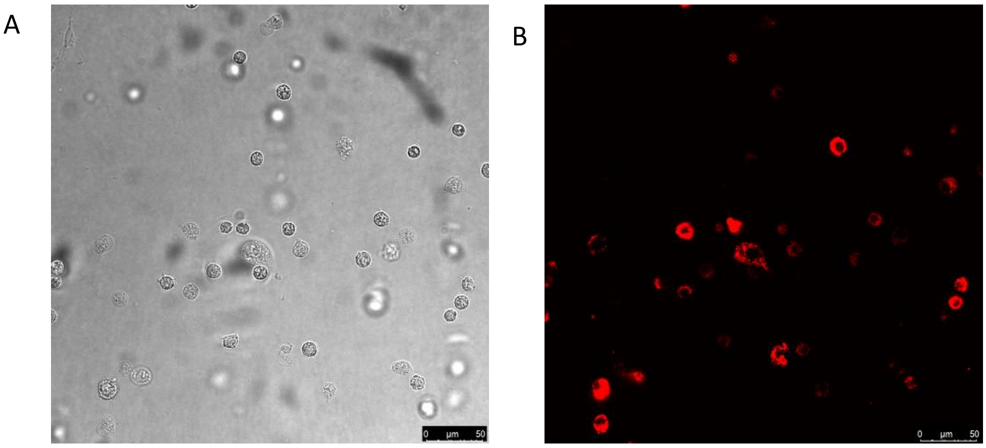

Supplementary Figure S1. Representative image of macrophage cells after uptake of *L. amazonensis* EVs stained with PKH26 (red marker). The uptake experiments were performed twice with similar results. (A) Phase contrast, and (B) fluorescence microscopy images demonstrating the uptake of labeled EVs.

Supplementary Table S1. Oligonucleotide primer sequences.

Target gene	Forward (5’ - 3´)	Reverse (5’ - 3´)
arg	GGAGAAGCTTGGATGGGATAC	GACCGATTCTGTCGCCATTA
gp63	CCGTCCTTCTCTAAAGGGTATTT	GTACAGCGAGGCGTTTATCT
hsp78	AGCGAACTGCTCAACCAA	AATCACCACGACATGACTATCC
lpg3	GTACTGCAAGAGAGCCGTATC	TCTCCACCTGCTTTCCATTC
ef1-a	ATGTGCGTGGAGGTGTTC	CGCTGCTCTCCTTCTTGTT
oligo-b	GGAAACCCGAACGAGTACAA	CCGCACTGGACCATGATATT
cpb	TAAGAAGCGCCACAGTAAGG	TTCTGCGTGTTGAGGAAGTAG
cpc	CCCGAACACCATCTACAATACC	GCCCTTGATGGAGTAAGATGAG
α-tub	AGCACACCGATGTTGCGACGAT	GATCAGGCGGTTCACGTTCGTGT
